# Effects of Thermal Damage on Impact Response Characteristics of High-Energy Propellants

**DOI:** 10.3390/polym16060748

**Published:** 2024-03-08

**Authors:** Fengwei Guo, Jianxin Nie, Suoshuo Zhang, Jiahao Liang, Rui Liu, Yu Zou, Yong Han

**Affiliations:** 1 State Key Laboratory of Explosion Science and Safety Protection, Beijing Institute of Technology, Beijing 100081, China; 2 601 Institute, The Sixth Academy of China Aerospace Science and Industry Corporation, Hohhot 010076, China

**Keywords:** high-energy propellant, thermal damage, microporous structure, impact, sensitivity

## Abstract

Thermal damage due to microstructure changes will occur in propellants under thermal stimulation. It can significantly affect the sensitization, combustion, and other properties of the propellant, which, in turn, affects the impact safety of the solid propellant rocket engine. A new component which uniformly heats the sample was designed to conduct the Lagrange test and EFP impact test at different temperatures. The thermal decomposition and damage characteristics of the propellant during the heating process were quantitatively analyzed. Additionally, the effects of ambient temperature on impact initiation and detonation growth of the high-energy propellant were elucidated at a mesoscopic level. The results showed that the porosity of the specimen increased by 0.89% under the thermomechanical mechanism, which was mainly characterized by interfacial de-bonding between the AP and the binder. The increase in thermal damage changed the hot spot reaction rate and significantly affected the growth process of propellant impact initiation. A method was proposed to systematically calibrate the reaction rate model for the propellant at different temperatures. The theoretical model parameters of the high-energy propellant at two typical temperatures were calibrated in this way. The critical shell thicknesses computed using LS-DYNA, which, for 20 and 70 °C, were obtained as 15 and 20 mm, respectively.

## 1. Introduction

Propellants are inevitably exposed to unexpected thermal stimuli during their life cycle, including fires or high-temperature environments. These stimuli can cause mesoscopic damage to propellants [1,2,3,4,5,6]. The internal damage of the propellants leads to a deterioration in the mechanical performance, resulting in a decreased strength and stiffness of the structure. The damage will further expand and gather under the influence of these factors, such as load and temperature, thereby affecting the sensitivity, combustion, and even explosive properties of the propellants [7,8,9,10]. To assess the safety and predict the life of propellants, it is of paramount importance to investigate the mechanisms of damage formation and ignition response of high-energy propellants at different temperatures.

A large number of studies have shown that the microstructure of explosives and propellants significantly changed under thermal stimulation [11,12,13]. The mechanical and combustion properties of energetic materials are significantly affected by thermal damage [11,14]. Tolmachoff et al. [15] analyzed the low-temperature decomposition of AP particles, and the morphology of porous ammonium perchlorate was observed using SEM. Tussiwand et al. [16] found that propellants became relatively brittle in low-temperature environments, exhibiting increased crack propagation rates compared to ambient conditions. The characteristics of crack propagation changed under low-temperature conditions, displaying AP particle fracture features, with no evident blunting process and a desensitized damage zone at the crack tip. Partom [17] demonstrated a significant relationship between the pressure generated during thermal stimulation and the plastic strain rate of LX-04, based on experiments conducted by Susan [18] and Steven [19]; these results helped to determine the ignition temperature threshold under thermal stimulation. Nair et al. [20] investigated the interaction of ammonium perchlorate with RDX, HMX, and PETN and the effect of the interaction on the thermal decomposition of ammonium perchlorate. The thermal degradation and decomposition of the propellant at different temperatures can improve the combustion rate [21,22]. These studies have shown that the damage of explosives and propellants significantly changed at different temperatures. But a quantitative description of thermal damage has not been provided. Hence, the quantitative characterization of thermal damage and the effects of thermal damage on the impact response characteristics of high-energy propellants should be further explained to better evaluate the safety of high-energy propellants.

In addition, properties such as the thermal decomposition and crystalline transformation of the energetic materials after heating can significantly affect its impact detonation characteristics [23,24]. Dallman et al. [25] used an improved wedge test and high-speed photography to investigate the effect of temperature on the shock sensitivity of PBX 9502 and LX-17. The pressure over particle (POP) velocity curves showed that the sensitivity of PBX 9502 at 252 °C remained lower than that of PBX 9501 at ambient temperature. The research of impact initiation characteristics of propellants generally adopts the Lagrange test, flyer plate impact test, gap test, and so on [26,27,28]. Urtiew et al. [29,30] performed flyer plate impact detonation experiments on LX-17 and other explosives under different temperatures. The results demonstrated that as the initial temperature of the tested explosives increased, the critical initiation pressure decreased and the shock wave sensitivity increased. Wang et al. [31] studied the thermal safety of a solid rocket motor with a complex charge structure and found that the heating rate has a dramatic effect on the ignition time. However, a quantitative description of thermal damage has not been provided. The mechanistic explanation of sensitivity and following the reaction at different temperatures are not clearly understood. Hence, the ignition mechanism of high-energy propellants at different temperatures needs to be further studied by means of experiments and simulations.

In summary, there are few studies on the quantitative characterization of damage and the effect of the thermal damage evolution of the ignition under combined thermal and impact insults. The mechanistic explanation of sensitivity and following the reaction at different temperatures are not clearly understood. In this study, a heating experimental setup was designed to investigate the influence of temperature on the thermal decomposition characteristics and microstructure of N15. Both the heating Lagrange test and cylinder test were designed to study the impact initiation law for N15 at two typical temperatures, and the model parameters of N15 were calibrated. This model can describe the impact initiation characteristics of propellants at different temperatures. The results of this study can provide references for the design and optimization analysis of the impact safety of propellants.

## 2. Quantitative Characterization of Thermal Damage in Propellants

### 2.1. Composition of the Propellant

N15 is a heterogeneous, high-energy propellant composed of octahydro-1,3,5,7-tetranitro-1,3,5,7-tetrazocine (HMX) particles (20–25%), ammonium perchlorate (AP) particles (45–55%), binder (17–25%), and aluminum powder (6–12%), with a mass density of 1.829 g/cm^3^.

### 2.2. Propellant Heating Test

#### 2.2.1. Test Setup

**Figure 1:**
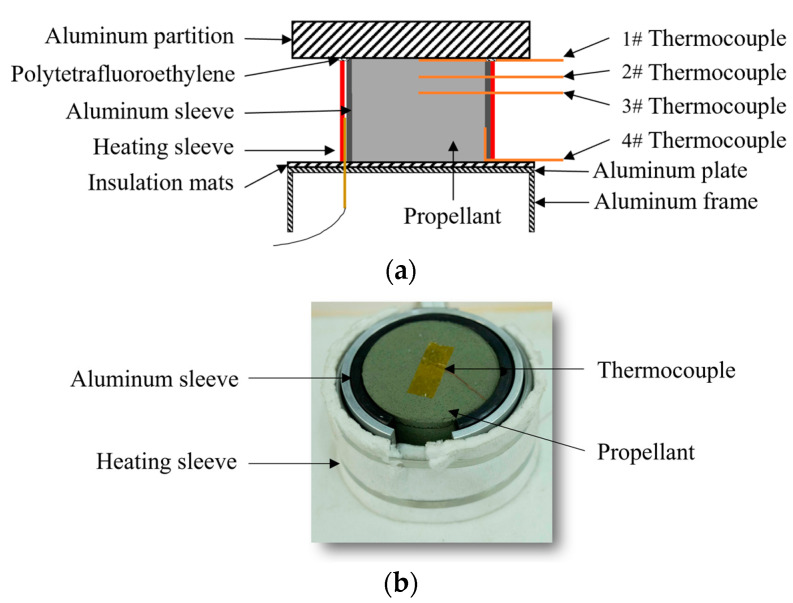
Propellant heating test setup. (**a**) Schematic diagram and (**b**) configuration diagram.

The heating test setup, shown in Figure 1, employed a heating sleeve to achieve uniform heating around the propellant and to reduce the heating time. The inner diameter of the heating sleeve was 60 mm. To accommodate the placement of temperature sensors for subsequent heating Lagrange testing, a 20 mm wide opening was cut on the side of the heating sleeve. Insulation cotton was wrapped around the bottom of the propellant and also around the opening of the heating sleeve to prevent heat dissipation; this would help reduce the temperature gradient within the propellant. Thermocouples for temperature measurements were attached at the top, 6 mm, 10 mm, and the side of the propellant.

The partition materials that are usually used are aluminum and organic glass. Owing to its poor thermal stability, organic glass softens at 40–50 °C. Therefore, aluminum was chosen as the material for the uniform heating of the propellant. However, as aluminum has a high thermal conductivity of 237 W·m^−1^·K^−1^, heat dissipation is likely to happen at the top of the propellant during the heating process. Polytetrafluoroethylene (PTFE) was added as thermal insulation between the aluminum partition and the propellant to reduce the heat dissipation and temperature rise at the top of the heating device. The PTFE has a low thermal conductivity of 0.25 W·m^−1^·K^−1^. At the same time, a 5 mm thick insulation pad was placed at the bottom of the propellant, to prevent heat loss from the bottom of the test setup as well. The validity of the heating test setup was verified using numerical simulations.

**Figure 2:**
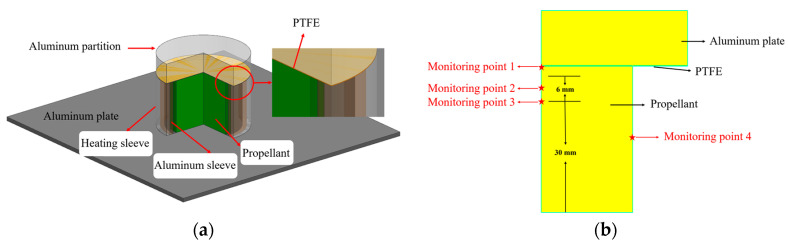
Propellant heating simulation model. (**a**) Physical model and (**b**) simulation model.

A physical model of the propellant heating simulation is shown in Figure 2a. During the heating, the aluminum sleeve was used to ensure the uniform heating of the propellant. The dimensions of the propellant charge were Φ50 × 40 mm, while those of the aluminum plate were Φ80 × 15 mm; the thickness of the PTFE film was 0.2 mm. Four temperature monitoring points were set inside the propellant, as shown in Figure 2b.

**Figure 3:**
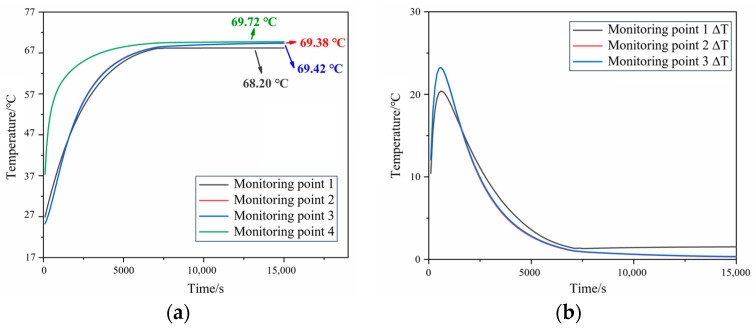
Simulation results of N15 during the thermal equilibrium process. (**a**) Internal temperature curves and (**b**) internal temperature difference curves.

Figure 3a illustrates the temperature curve of the propellant during the heating process towards a thermal equilibrium at 70 °C. Figure 3b presents the temperature difference within the propellant during the heating process, where ΔT is the temperature difference between monitoring points 1–3 and 4 on the wall surface. It was observed that monitoring point 4, being close to the heated wall surface, exhibited a rapid temperature rise; in contrast, monitoring point 3, located near the center of the charge, experienced a slower temperature increase at the beginning of heating. During the heating process, the temperature difference within the propellant initially increased and then gradually decreased. With the increase in the time, the temperatures at all the monitoring points approached 70 °C, with temperature differences being smaller than 5 °C. Numerical simulation results show that the aluminum and PTFE can effectively reduce heat dissipation and can achieve the uniform heating of the propellant.

#### 2.2.2. Propellant Heating Test Results

**Figure 4:**
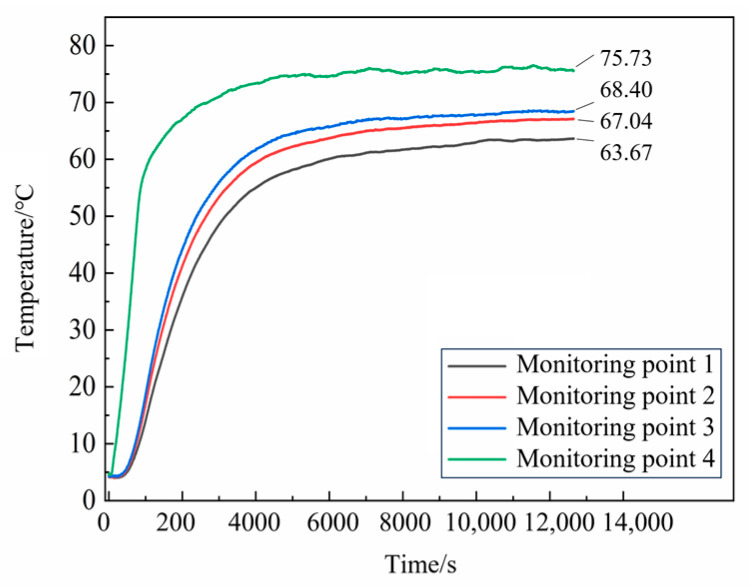
Temperature curves of N15 from the heating test.

A heating test was conducted on N15 at 70 °C. To ensure that the propellant reached the desired temperature, the constant heating temperature was raised by 10 °C. The temperature–time curves from the test at different locations within the propellant are shown in Figure 4.

From Figure 4, it can be seen that the temperature on the surface of the propellant increased rapidly during the heating process, while the temperature in the interior increased gradually. After approximately 5000 s of heating, the internal temperature of the propellant stabilized. At 12,000 s, the temperatures measured by thermocouples 1, 2, 3, and 4 were 63.42, 66.97, 68.36, and 75.88 °C, respectively. The temperature differences from 70 °C at each measurement location were 9.4%, 4.3%, 2.3%, and 8.4%, respectively, all of which were less than 10%.

### 2.3. Thermal Decomposition Characteristics of N15

**Figure 5:**
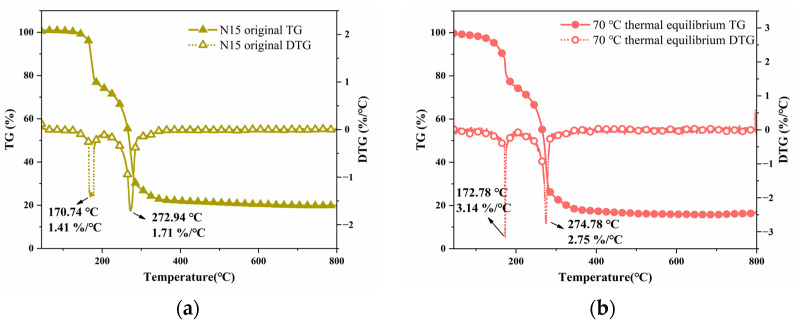
TG and DTG curves of the propellant at different temperatures. (**a**) TG and DTG curves of the propellant at 20 °C and (**b**) TG and DTG curves of the propellant at 70 °C.

Figure 5 presents the TG and DTG curves of both the unheated and heated (towards thermal equilibrium at 70 °C) propellant samples. It can be observed that the thermal weight loss process of the unheated sample consisted of two stages. The first stage, within the range of 110–205 °C, exhibited a sharp peak at 170.74 °C, with a weight loss of approximately 25.90% and a maximum weight loss rate of 1.41%/°C. This stage primarily involved the decomposition of the binder in the propellant. The second stage, within the range of 205–360 °C, demonstrated a weight loss of approximately 51.67%, with a sharp peak at 272.94 °C and a maximum weight loss rate of 1.71%/°C. This stage mainly involved the decomposition of AP and HMX. The remaining residue accounted for approximately 22.43%.

The thermal weight loss process of the heated propellant sample, during the process of reaching the thermal equilibrium at 70 °C, also consisted of two stages. The first stage, within the range of 110–205 °C, exhibited a sharp peak at 172.78 °C, with a weight loss of approximately 25.03% and a maximum weight loss rate of 3.14%/°C. This stage primarily involved the decomposition of the binder chain in the propellant. The second stage, within the range of 205–365 °C, demonstrated a weight loss of approximately 58.64%, with a sharp peak at 274.78 °C and a maximum weight loss rate of 2.75%/°C. This stage mainly involved the decomposition of AP and HMX. The remaining residue accounted for approximately 16.33%.

It can be seen that the thermal decomposition processes of both the unheated and heated (thermal equilibrium at 70 °C) propellant samples consisted of two stages. However, after heating the propellant, the peak temperatures of the first and second weight loss stages increased, as heating the propellant resulted in the partial decomposition of the binder in the mixture, leading to higher decomposition peak temperatures. The heat accumulation of the heated propellant is more rapid than that of the unheated propellant, due to its higher peak temperatures of the thermal decomposition stages. The size and number of pores inside the propellant continued to increase. Under the action of the impact wave, the air inside the pore is more easily compressed than the explosive particles, which produces a large amount of heat. It increases the hot spot reaction rate inside the propellant, resulting in an increase in the energy release of the N15 propellant.

### 2.4. Gaseous Products Analysis

**Figure 6:**
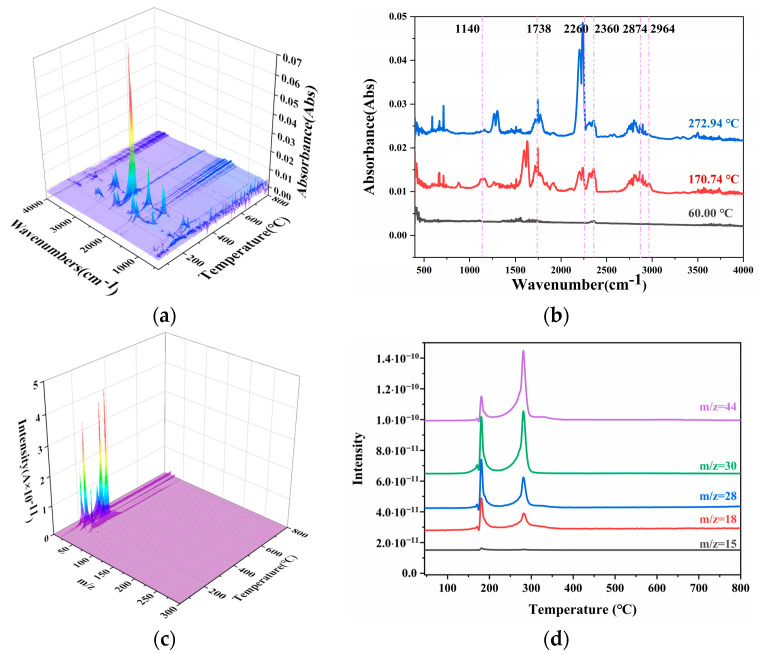
The FTIR and MS curves of N15 at 20 °C. (**a**) The FTIR curves of N15, (**b**) the absorbance distribution curves of N15, (**c**) the MS curves of N15, and (**d**) the intensity curves of N15.

**Figure 7:**
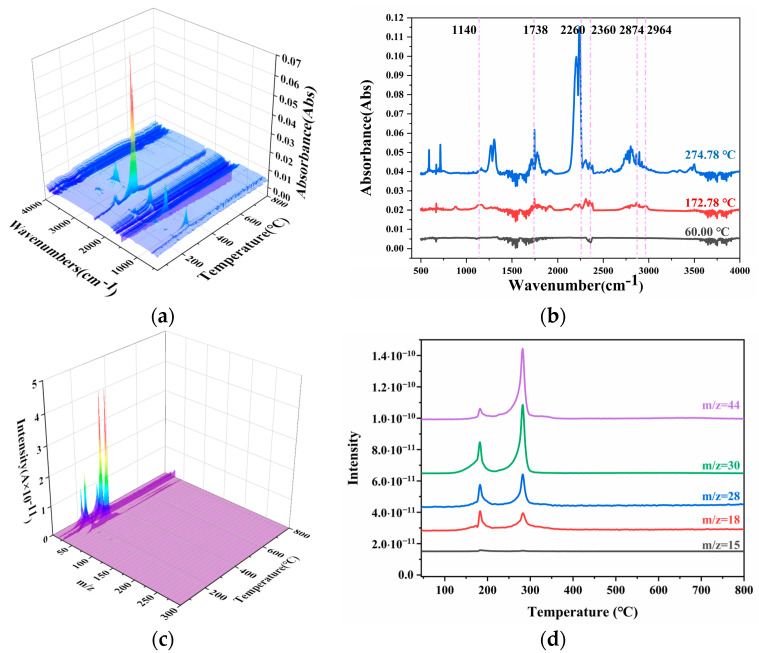
The FTIR and MS curves of N15 at 70 °C. (**a**) The FTIR curves of N15, (**b**) the absorbance distribution curves of N15, (**c**) the MS curves of N15, and (**d**) the intensity curves of N15.

Figure 6 and Figure 7 display the Fourier transform infrared spectroscopy (FTIR) and mass spectroscopy (MS) curves of both the unheated and heated (at 70 °C) propellant samples. It can be observed that the FTIR curve at the peak temperature of the propellant weight loss corresponded to the following groups: 2874–2964 cm^−1^ for C-H (2964 cm^−1^ specifically corresponded to the asymmetric stretching vibration peak of C-H in -CH_3_, and 2874 cm^−1^ corresponded to the symmetric stretching vibration peak of C-H in -CH_3_), 2260 cm^−1^ for N_2_O, 1738 cm^−1^ for the stretching vibration peak of C=O, and 1140–1178 cm^−1^ for the stretching vibration peak of C-O. When this was compared with the MS spectrum, it could be determined that the gaseous products in the first weight loss stage of the propellant were small amounts of -CH_3_, CO, CO_2_, N_2_O, and NO. In the second weight loss stage, the concentration of N_2_O significantly increased, while that of CO_2_ and CO decreased considerably. For the heated (at 70 °C) propellant samples, the concentrations of CO_2_, CO, and N_2_O all significantly increased in the second weight loss stage.

From the curves in Figure 6 and Figure 7, it can be observed that during the process of reaching a thermal equilibrium at 70 °C, the characteristic absorption peaks of the propellant’s first weight loss stage all decreased, as did the C-O stretching vibration peak at 1140–1178 cm^−1^. It can be seen that with increasing temperature, the gaseous products containing -CH_3_, CO, CO_2_, N_2_O, and NO began to volatilize from the propellant. As the temperature continued to rise, only a few volatile substances remained in the propellant. When heated again, the substances that had not completely volatilized in the first stage continued to evaporate, resulting in a slight increase in the peak temperature of the first weight loss stage.

### 2.5. Micro-CT Characterization of N15

**Figure 8:**
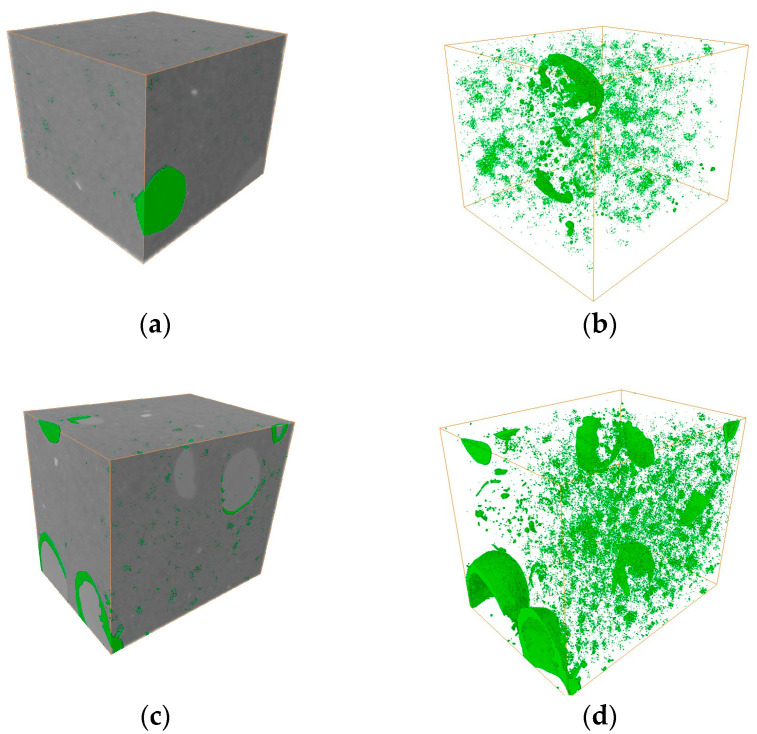
Micro-CT of N15 at different temperatures. (**a**) The 3D view of N15 at 20 °C, (**b**) the pore distribution of N15 at 20 °C, (**c**) the 3D view of N15 at 70 °C, and (**d**) the pore distribution of N15 at 70 °C.

The 3D morphological information of the propellant samples and internal damage after different temperature heat treatments was obtained through Micro-CT scanning and data processing. Figure 8 shows the Micro-CT results of the propellant samples. The light gray, dark gray, white, and green areas represent the binder matrix, HMX, AP particles, and internal pores within the explosive, respectively.

In the propellant sample, the main pores and defects between the particles were fine irregular shapes at 20 °C. A slight interfacial de-bonding between the explosive particles and binder, as well as some internal pores within the explosive particles, could be noticed. After the heat treatment at 70 °C, the degree of de-bonding between the explosive particles increased significantly and the damage in the sample could mainly be characterized by the interfacial de-bonding. The de-bonding was primarily governed by thermomechanical mechanisms, such as the anisotropic thermal expansion of the AP particles, and the difference in the thermal expansion coefficients between the AP particles and the binder.

#### 2.5.1. Porosity

**Figure 9:**
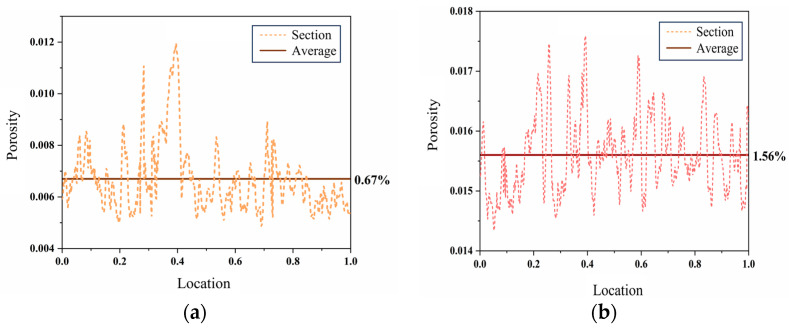
The porosity of N15 at different locations. (**a**) The porosity of N15 at 20 °C and (**b**) the porosity of N15 at 70 °C.

Based on the 3D damage characteristics of the N15 samples, the porosity of their 2D CT slices was calculated to investigate the evolution of porosity with temperature. The distribution curves of porosity along the slicing positions (the slicing position at the axial center of the sample was taken as the origin and positive upward direction) for the samples after the heat treatment at different temperatures are shown in Figure 9. It can be observed that the porosity of the unheated propellant was 0.67%; this was mainly caused by defects in the manufacturing process. At 70 °C, during the process of reaching a thermal equilibrium, the porosity of the propellant was 1.56%, and most of the pores existed in the binder matrix. This was because the decomposition temperatures of HMX and AP are higher than 70 °C and, hence, these constituents had not decomposed yet; in contrast, the decomposition temperature of the binder is lower and, hence, it underwent its initial decomposition. This was consistent with the observed thermal decomposition characteristics of the propellant. Most of the pores were located at the interfaces between the binder and HMX/AP particles, indicating that the decomposition occurred first in these regions.

#### 2.5.2. Pore Size Distribution

A 3D pore structure model was used to analyze the distribution of pore sizes (represented by pore volume) in the propellant samples after heat treatments at different temperatures. The numbers of pores with different sizes were statistically analyzed and studied and are listed in Table 1. At 20 °C, the pores had relatively small diameters, with the majority falling within the range of 0–1 μm^3^ and reaching a count of 14,250. As the temperature increased, most of the pores in the sample fell within the range of 1–10 μm^3^, reaching a count of 16,834. At 70 °C, the number of pores in the 0–1 μm^3^ range decreased by 63% compared to those in the sample at 20 °C. This is due to the fact that the heat treatment causes interfacial de-bonding to occur between the particles and the binder, and these small pores merge to form large pores.

**Figure 10:**
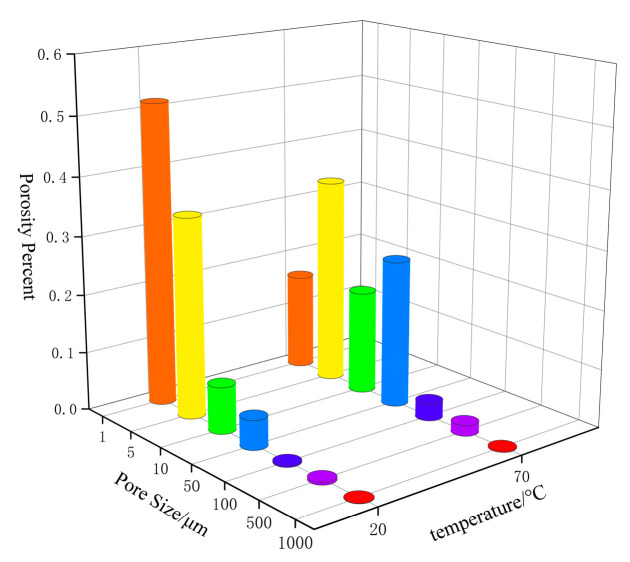
Pore size distribution of N15 at different temperatures.

The pores gradually increase under thermal stimulation. The results of the internal pores of the specimen at different temperatures are shown in Figure 10. The pore volume corresponding to each pore size range was calculated and normalized to obtain the pore volume percentage, which represents the pore size distribution. At 20 °C, the majority of pore sizes were within the range of 0–10 μm^3^, with a size distribution of 94.1%. In the sample at 70 °C, the pore sizes had a wider range, mainly within the 0–100 μm^3^ range, with a cumulative size distribution of 97.8%. The proportion of large pores (10–100 μm^3^) in the sample heated at 70 °C was 5.2 times more than that in the sample heated at 20 °C. As the temperature increases, the number of large pores inside the sample increases and the percentage of large-size pores increases. With the increase in porosity and the pore size distribution, the yield strength and compressive strength of the material will decrease. Therefore, the propellant is more susceptible to plastic deformation when it is subjected to impact loading. Plastic deformation generates a lot of heat and the heat and gas products are released by the decomposition of oxidizer particles when the temperature rises to a certain level. The transition rate of the propellant from deflagration to detonation is accelerated for this reason, which reduces the safety of the propellant.

#### 2.5.3. Specific Surface Area

Based on the established 3D pore structure model, the surface areas and specific surface areas of the pores were calculated through image processing, and the results are presented in Table 2. After reaching thermal equilibrium at 70 °C, more pores were formed within the sample, leading to an increased pore surface area and specific surface area. At 70 °C, the binder decomposed at a low temperature, while the decomposition temperatures of HMX and AP were high and, hence, their decomposition had not yet started at 70 °C. The pore damage structure caused by de-bonding between the HMX/AP particles and the binder led to an increase in the pore surface area and specific surface area. The specific surface area increased by a factor of 1.77. The larger the porosity and specific surface area of the propellant, the larger the combustion area after ignition. This led to a faster combustion rate and higher rate of increase in pressure.

#### 2.5.4. Sphericity

The geometric morphology of pores is crucial for the mechanical and combustion performance of the propellant. Long pores provide channels for product gases and combustion flames to enter the explosive matrix, thereby causing a transition from a laminar to a convective combustion within the propellant. Sphericity (*S*) is often used to characterize the geometric shape of pores and is defined as the ratio of the surface area of a sphere with the same volume as the given pore to the surface area of the pore itself; it can be calculated using the formula:

(1) $$S=\frac{\pi^{1/3}{\left(6V\right)}^{2/3}}{A}$$

where *V* and *A* are the volume and surface area of the pore, respectively. A value of 1 for sphericity *S* indicates that the pore shape is spherical. A small value of *S* indicates a relatively irregular or elongated shape.

**Figure 11:**
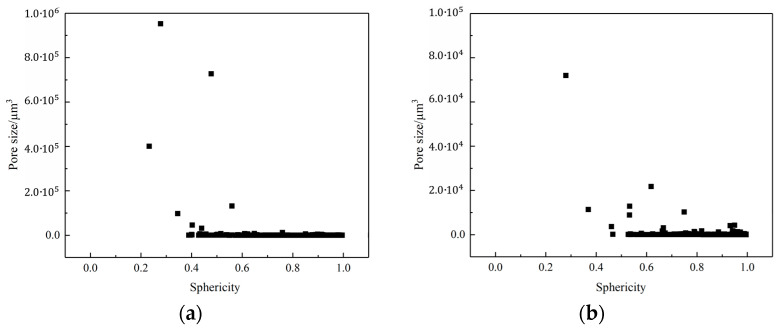
Sphericity of pores at different temperatures. (**a**) The sphericity of pores at 20 °C and (**b**) the sphericity of pores at 70 °C.

Figure 11 shows the sphericity of pores at different temperatures. At both temperatures, sphericity of the pores was inversely correlated to the pore volume. Smaller pores tend to have a larger sphericity, while larger pores tend to have a smaller sphericity. In the propellant sample at 20 °C, the sphericity of the pores lies between 0.4 and 1.0, whereas, in the sample heated at 70 °C, the sphericity of the pores lies between 0.5 and 1.0; this implies that the pores had a more spherical shape at 70 °C and this was not conducive to the transition from a laminar to a convective combustion within the propellant.

#### 2.5.5. Mechanism of Damage

**Figure 12:**
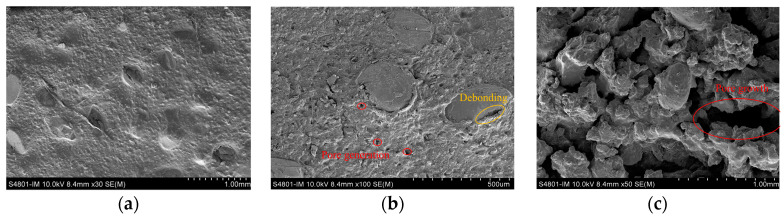
SEM of N15 samples at different temperatures. (**a**) 20 °C, (**b**) 70 °C, and (**c**) 100 °C.

The scanning electron microscopy (SEM) results of the propellant samples at different temperatures are shown in Figure 12. At 20 °C, the components within the propellant were tightly bonded, and no evident pores were formed. At 70 °C, an interfacial de-bonding between the AP particles and the binder occurred and a large number of pores were present within the binder. At 100 °C, the size and number of pores inside the propellant continued to increase and the interfacial de-bonding between the AP particles and the binder intensified. Under thermal stimulation, de-bonding occurred at the interface between the AP particles and the binder, especially for the larger AP particles, while no trans-crystalline fracture or fragmentation occurred within the AP particles.

The critical stress for de-bonding failure at the particle–binder interface ($\sigma_{d})\;$under thermal stimulation is given in Equation (2) [32]:

(2) $$\sigma_{d}^{2}=\frac{4E\gamma \left(1+v\right)}{9r{\left(1-v\right)}^{2}}$$

where *E* is the Young’s modulus of the binder; v is the Poisson ratio; γ is the surface energy of the interface; and r is the radius of the spherical particle. If the stress on the explosive particles exceeded $\sigma_{d}$, de-bonding would occur at the particle–binder interface.

According to Equation (2), $\sigma_{d}$ is inversely proportional to the particle radius r. Therefore, larger particles are more prone to de-bonding compared to smaller particles and this is consistent with the SEM observations, presented in Figure 12.

As the heat treatment temperature increases, the thermal stress in the specimen rises. The critical de-bonding stress is reached at the interface between the explosive particles of different sizes and the binder. It leads to the gradual de-bonding of AP particles from large to small sizes and the area within the sample carrying thermal stresses decreases. It accelerates the development of de-bonding. In summary, the experimental results with N15 demonstrated that the damage increases with increasing temperature. Most of the damage was manifested as small but discrete holes and not as interconnected pores. Thus, it was specifically manifested as interfacial de-bonding between the AP particles and the binder.

**Figure 13:**
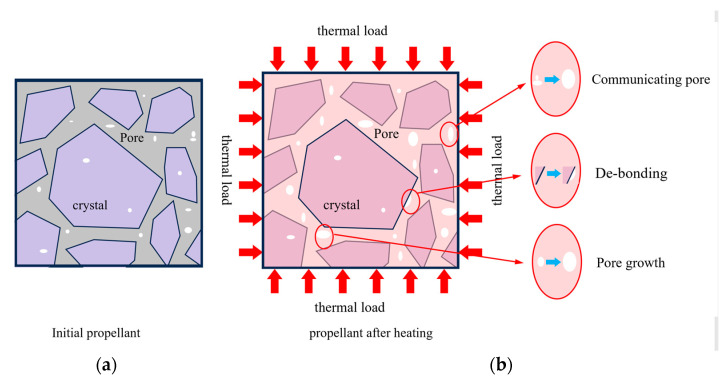
Schematic of damage mechanism. (**a**) Initial propellant and (**b**) propellant after heating.

A schematic of the damage mechanism of the propellant is shown in Figure 13. In the initial propellant sample, the main pores and defects between the particles were fine irregular shapes. After the heat treatment, the degree of de-bonding between the explosive particles increased significantly and the damage in the sample could mainly be characterized by the interfacial de-bonding, pore growth, and the small pores penetrating to form communicating pores.

## 3. Reaction Model of N15 at Different Temperatures

At 70 °C, the thermal damage inside the samples increased, resulting in an increased reaction rate of the hot spot and a reduced impact initiation threshold of the high-energy propellant. Therefore, based on the uniform heating test device, which was described in Section 2, a cylinder test and heating Lagrange test under two typical temperatures were conducted, and the parameters of the ignition and growth model of N15 at both 20 and 70 °C were calibrated based on the experimental results.

### 3.1. Equation of State of Reactants and Detonation Products

#### 3.1.1. Cylinder Test

**Figure 14:**
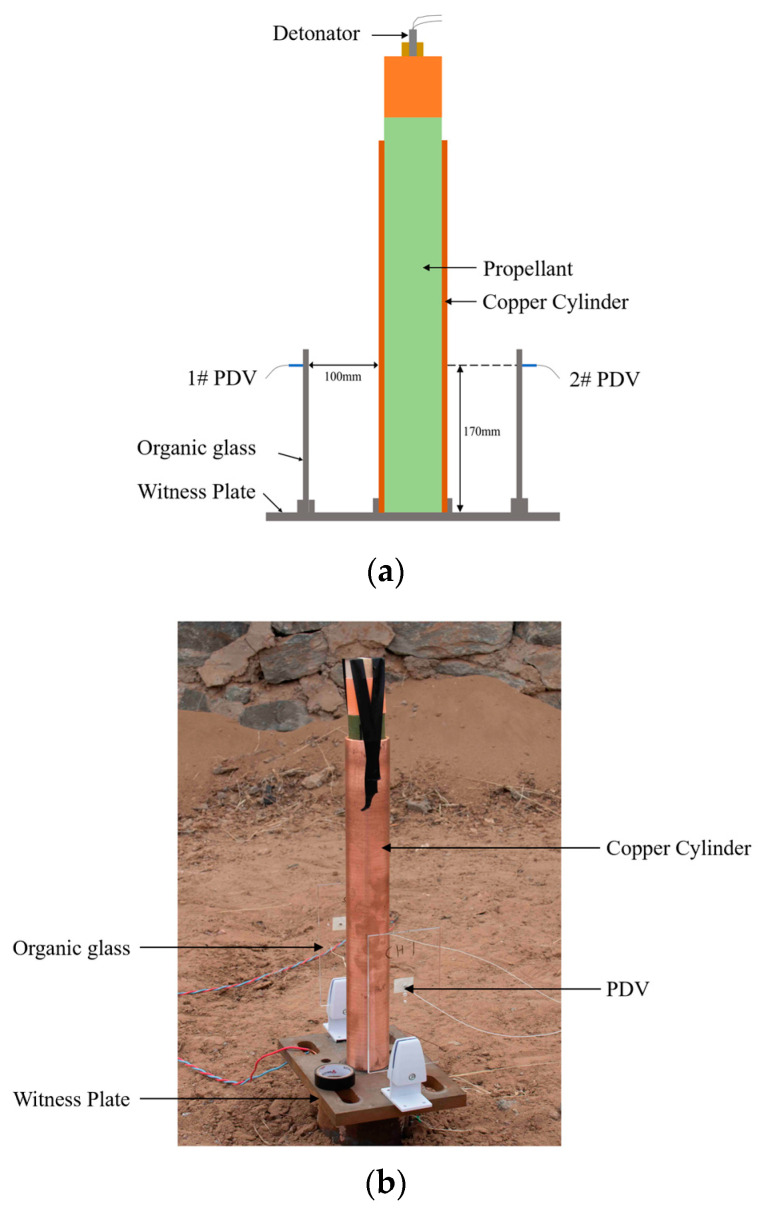
The schematic diagram of the cylinder test. (**a**) Schematic diagram and (**b**) configuration diagram.

A schematic diagram of the cylinder test is shown in Figure 14. The inner diameter of the cylinder is 50 mm, the wall thickness is 5 mm, and the length is 500 mm. The cylinder test device consisted of a detonator, a booster explosive, a copper cylinder, a propellant, a PDV, and a witness plate. The Φ50 mm cylinder was placed vertically on the support frame and the expansion velocity of the cylinder was measured at a height of 200 mm using a laser interferometer.

The wall velocity of the cylinder was measured using the PDV, whose probe was attached to a highly transparent organic glass plate, and emitted a laser toward the cylinder wall. The laser was reflected back to the PDV probe after being irradiated on the cylinder wall. Owing to the Doppler effect, the frequency of the laser signal changes with the variation of the cylinder expansion velocity, which can be calculated based on the frequency change of the reflected laser signal after interference, thus allowing for a contactless measurement. Traditional cylinder tests use high-speed photography to measure the wall velocity; however, the PDV method has advantages, such as high time resolution, safety, and reliability. Additionally, the wall velocity obtained from high-speed photography is in Eulerian coordinates, while the velocity obtained from the PDV method is in Lagrangian coordinates; this is consistent with the velocity calculated during the calibration process.

#### 3.1.2. Test Results

**Figure 15:**
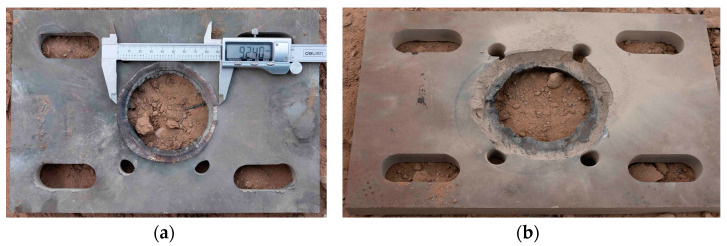
Shape of the witness plate after the test. (**a**) Front of the witness plate and (**b**) back of the witness plate.

Photographs of the witness plate after the test are shown in Figure 15. A 20 mm thick witness plate made of 45 steel was perforated, and the diameter of the perforations reached 92.4 mm, indicating the occurrence of a detonation reaction in N15.

#### 3.1.3. Method of Parameter Calibration

The Jones–Wilkins–Lee (JWL) [33] equation of state for detonation products is given as:

(3) $$p=A\left(1-\frac{\omega }{R_{1}\bar{V}}\right)e^{-R_{1}\bar{V}}+B\left(1-\frac{\omega }{R_{2}\bar{V}}\right)e^{-R_{2}\bar{V}}+\frac{\omega E}{\bar{V}}\;\;$$

where p is the product pressure; $\bar{V}=V/V_{0}=\rho_{0}/\rho$ is the relative specific volume of the product; $\rho_{0}$ is the initial density of the explosive; and *A*, *B*, *C*, *R*_1_, *R*_2_, and *ω* are constants.

**Figure 16:**
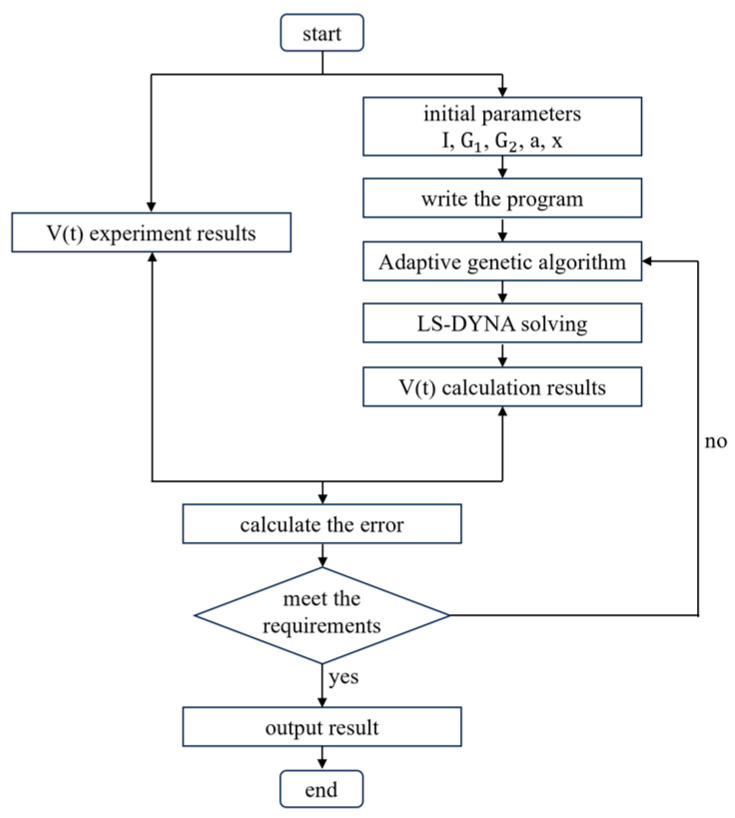
Flow chart of parameter calibration.

The six coefficients in the JWL equation needed to be calibrated. First, a simulation model for the cylinder test was established. Then, based on the difference between the calculated and experimental data, a genetic algorithm to automatically calibrate the parameters was used to adjust the JWL equation parameters. The parameter calibration process is illustrated in Figure 16.

#### 3.1.4. Results of Parameter Calibration

**Figure 17:**
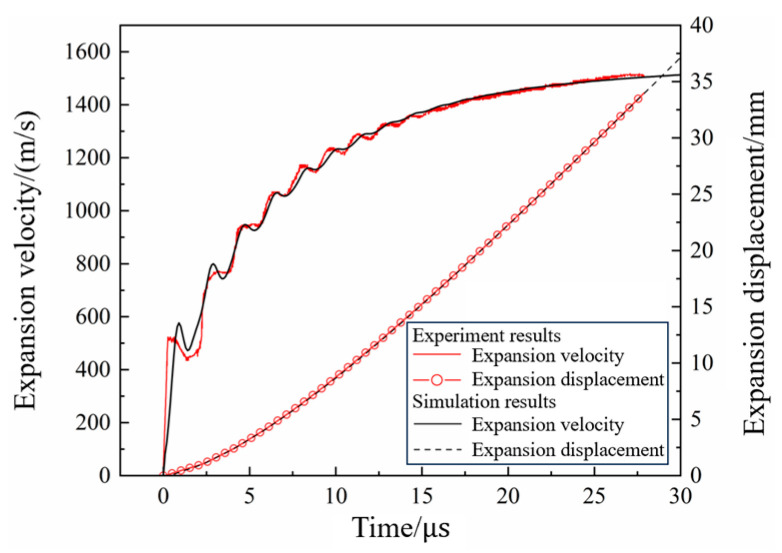
Wall expansion curves of cylinder test.

In the cylinder test, the calculated results of the wall expansion velocity and displacement curves were compared with the experimental results, as shown in Figure 17. It can be seen that the calculated and experimental results were in good agreement. After segmenting the linear interpolation of the velocity curve, the error between the simulated and the experimental results was calculated using the least squares method; this yielded an error of 3.67%, which indicates that the calibrated JWL equation parameters could effectively describe the process of metal acceleration driven by the detonation of N15. The Gurney velocity of the N15 high-energy propellant is calculated as 2469 m/s. The calibrated JWL equation parameters of N15 are presented in Table 3.

During the heating process, the thermal energy mainly caused a thermal expansion and thermal decomposition-led gas generation in the propellant; this resulted in changes in the microstructure of the propellant and affected its impact initiation behavior. Additionally, the influence of thermal expansion and decomposition on the component content of the propellant was minimal. Therefore, it was assumed that the detonation energy of N15 at 70 °C remained unchanged. The same equation of state parameters of detonation products was used for N15 at both 20 and 70 °C.

#### 3.1.5. Equation of State of Unreacted Propellant

The JWL equation of the state of the unreacted propellant describes the thermodynamic parameters of the propellant during the impact loading and is usually obtained through experiments or theoretical calculations. However, experimentally obtaining the JWL equation parameters of the unreacted propellant is complicated and expensive. Therefore, in this study, a theoretical calculation method was used to determine these parameters.

Su et al. [34] proposed a theoretical calculation method based on the superposition principle. This method calculates the shock wave constants based on the component content and properties of each component. According to the superposition principle, the formulae for calculating the shock wave constants of a mixture containing two components, 1 and 2, are given in the following equations:

(4) $$a_{12}=\frac{a_{1}a_{2}\left[\rho_{1}\left(1-x\right)+\rho_{2}x\right]}{a_{1}\rho_{1}\left(1-x\right)+a_{2}\rho_{2}x}\;\;$$

(5) $$b_{12}=\frac{b_{1}b_{2}\left[\rho_{1}\left(1-x\right)+\rho_{2}x\right]}{b_{1}\rho_{1}\left(1-x\right)+b_{2}\rho_{2}x}$$

where $a_{12}$ and $b_{12}$ are the shock wave constants of the mixture; $\rho_{1}$ and $\rho_{2}$ are the densities of components 1 and 2, respectively; $a_{1}$ and $b_{1}$ are the shock wave constants of component 1; $a_{2}$ and $b_{2}$ are the shock wave constants of component 2; and x is the content of component 1.

Using Rankine–Hugoniot equations, the *D*–*u* relationship is converted into a *P*–*V* relationship. The form of the equations is as follows:

(6) $$P=\rho_{0}\left[a+\left(\frac{ab\left(V-1\right)}{b-1-bV}\right)\right]\left[\frac{a\left(V-1\right)}{b-1-bV}\right]$$

**Figure 18:**
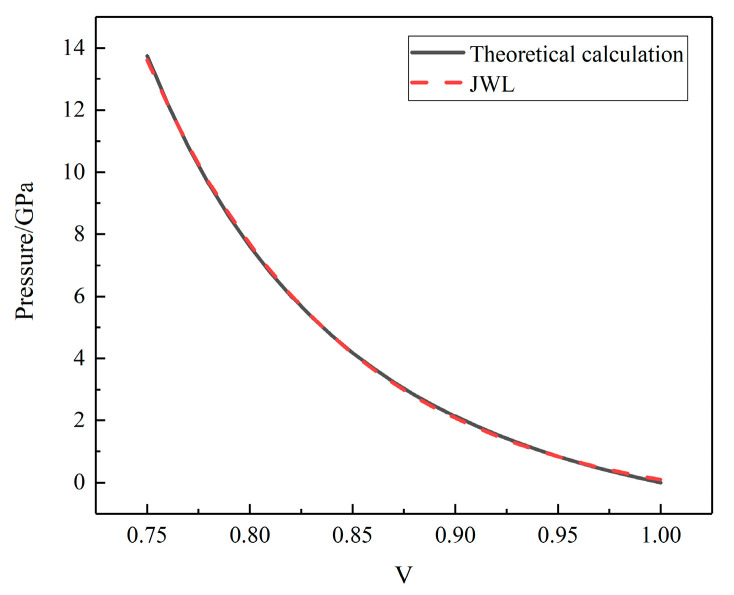
The P–V relationship of unreacted N15.

By combining Equations (4)–(6), the parameters of the JWL equation for the unreacted propellant can be determined. The fit of the *P–V* relationship obtained from the JWL equation is shown in Figure 18. The fitted parameters were the JWL equation parameters of the unreacted propellant, as shown in Table 4.

### 3.2. Reaction Rate Equation of N15

#### 3.2.1. Heating Lagrange Test Device

**Figure 19:**
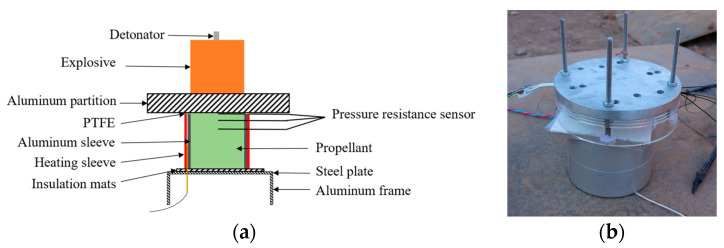
Schematic of heating Lagrange test device. (**a**) Schematic diagram and (**b**) configuration diagram.

Based on the experimental device for uniformly heating propellant samples, whose design was presented in Section 2, a heating Lagrange test device was designed as shown in Figure 19. This device mainly consisted of a detonator, aluminum partition, heating sleeve, propellant sample, pressure sensor, and thermocouples.

#### 3.2.2. Heating Lagrange Test Results

**Figure 20:**
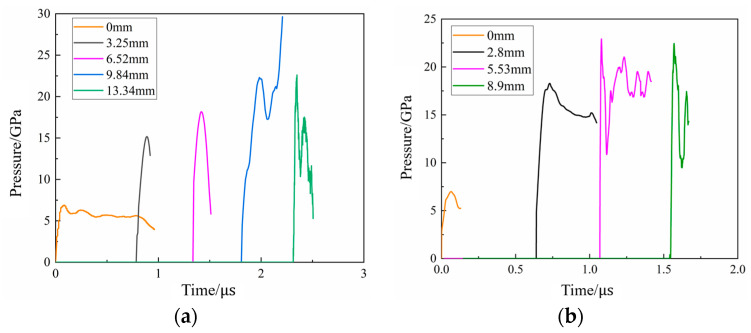
Pressure curves of the Lagrange test at different temperatures. (**a**) 20 °C and (**b**) 70 °C.

Impact initiation tests were performed on N15 at 20 °C and 70 °C with an aluminum spacer of 15 mm thickness. The pressure curves of the N15 propellant are shown in Figure 20. From Figure 20b, it can be seen that when N15 was heated to 70 °C and subjected to impact initiation, the peak pressure at a position of 2.8 mm reached 18.3 GPa. The peak pressures at positions of 5.53 and 8.9 mm are 22.9 and 22.4 GPa, respectively, and the velocity of the pressure wave between the two measurement positions is 7020 m/s, indicating that a stable detonation had occurred at 5.53 mm. The detonation pressure and velocity obtained at both 20 and 70 °C were basically the same. The detonation growth distance of N15 at 70 °C is between 2.8 and 5.53 mm, which is significantly shorter than that at 20 °C, indicating that the detonation growth distance is reduced, while the detonation growth velocity increased at 70 °C.

The thermal expansion occurred at 70 °C. Propellant density decreases, propellant impedance decreases, and propellant expansion increases the number of its internal pores. Under the action of the impact wave, the air inside the pore is more easily compressed than the explosive particles, which produces a large amount of heat. It increases the hot spot reaction rate inside the propellant, resulting in an increase in the reactivity of the N15 propellant. The growth distance of detonation is significantly shortened and the growth rate is accelerated.

#### 3.2.3. Calibration of Reaction Rate Equation Parameters

##### Calibration Method

The impact initiation process and shock-to-detonation (SDT) of the propellant can be analyzed using the classical hot spot theory. Lee and Tarver [35] proposed the ignition growth model in 1980. The reaction rate equation of the ignition growth model consists of three terms: (1) the ignition term, which represents the relationship between the explosive fraction and impact compression; (2) the slow growth term, which describes the gradual combustion propagation inward or outward from the ignition location; and (3) the fast completion term, which represents the rapid decomposition reaction of the unreacted explosive at high temperatures and pressures. The speed of this transition is related to the pressure. The reaction rate equation can be expressed as:

(7) $$\frac{dF}{dt}=I{\left(1-F\right)}^{b}{\left(\frac{\rho }{\rho_{0}}-1-a\right)}^{x}+G_{1}{\left(1-F\right)}^{c}F^{d}P^{y}+G_{2}{\left(1-F\right)}^{e}F^{g}P^{z}$$

where F is the reaction degree; t is the time; ρ is the density; $\rho_{0}$ is the initial density of the propellant; and I, $G_{1}$, $G_{2}$, a, b, x, c, d, y, e, g, and z are constants to be determined.

A simulation model for the heating Lagrange test was established. Based on the pressure curves obtained from this test, a nonlinear dynamics simulation and a genetic algorithm calibration procedure were used to minimize the error between the calculated pressure curve and experimental data. This iterative optimization would lead to the calibrated parameters of the ignition growth model.

##### Parameter Calibration

**Figure 21:**
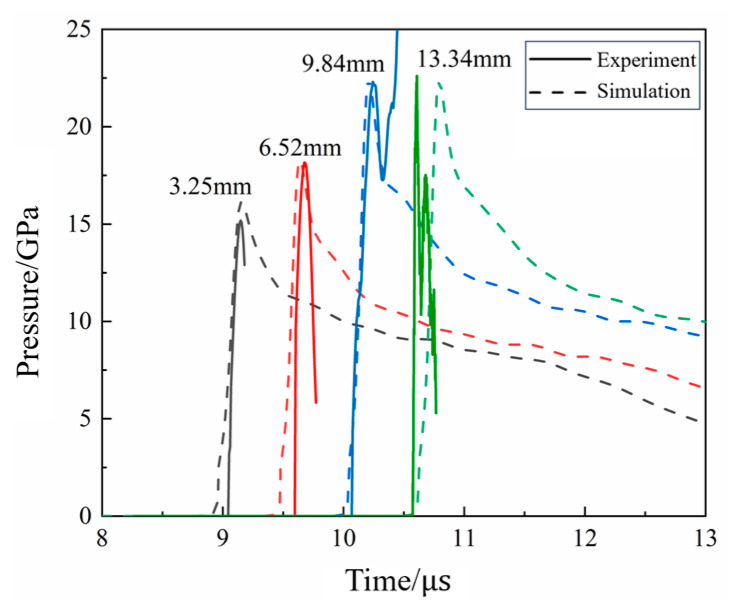
The simulation and experimental results of N15 at 20 °C.

Figure 21 compares the experimental measurements and simulation results of N15 at 20 °C during the impact initiation. It can be observed that the simulation results for the pressure growth inside N15 were in good agreement with the experimental results.

Table 5 compares the measured and calculated peak pressures at the monitoring points. The calculated values of the pressure during the impact initiation process of N15 were in good agreement with the experimental values. This indicates that the ignition and growth model parameters were successfully calibrated. The final calibrated model parameters of N15 at 20 °C are listed in Table 6.

The initiation of the propellant is controlled by the hot spot ignition process and the subsequent combustion reaction process. The hot spots are formed due to the compression of air inside the propellant, generating heat under the action of external loads. As the temperature increases, the thermal expansion of the specimen leads to a decrease in propellant density and the increase in the number of pores. The air inside the pore is more easily compressed than the explosive particles. So, the number of potential hotspots increases the hotspot reaction rate. It reduces the impact initiation threshold of high-energy propellants. Therefore, thermal damage was closely related to the ignition term of the ignition growth model. By adjusting the value of parameter *G*_1_, the chemical reaction process of the propellant at different temperatures can be accurately described.

**Figure 22:**
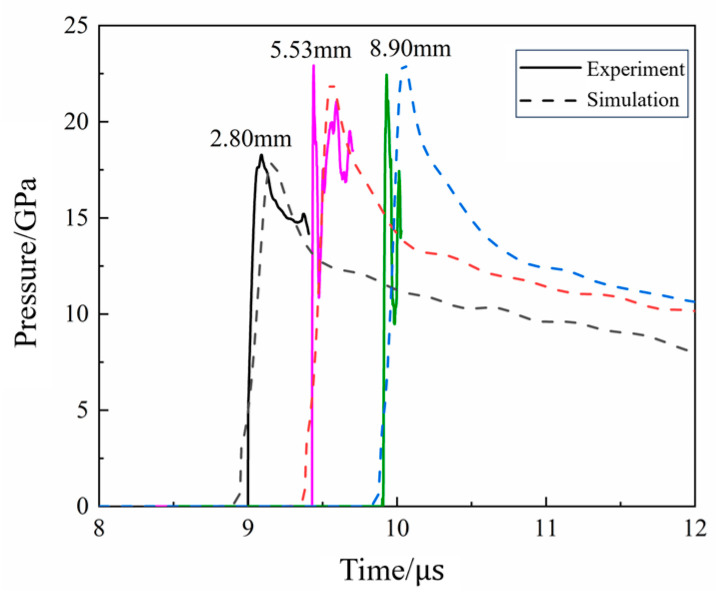
The simulation and experimental results of N15 at 70 °C.

For the calibration of the reaction rate equation parameters at different temperatures, a set of state equation parameters based on the Lagrange test results were calibrated at a reference temperature and the value of parameter $G_{1}$ was adjusted to obtain the ignition-growth model parameters at other temperatures [36]. Therefore, by adjusting the value of $G_{1}$ in the ignition growth model, the model parameters of N15 at 70 °C were obtained. Where $G_{1}$ = 377.23 Mbar^−2^·μs^−1^, the simulation and experimental results of N15 at 70 °C are shown in Figure 22. Furthermore, a comparison of the experimental and simulated peak pressures at the monitoring points is presented in Table 7. It can be seen that the simulated and experimental internal pressure fields of the propellant at 70 °C were in good agreement with each other, indicating that the calibrated parameters could describe the impact initiation behavior of N15 under different temperature conditions.

The comparison of each experiment with the numerical simulation results is shown in Figure 14, Figure 15, Figure 18 and Figure 19 and Table 5 and Table 7. The maximum error between the experimental and numerical simulation results of pressure was 6.25%, while the minimum error was 0.22%. The main reason for the errors included the measurement error of the experimental system mainly from the 2% sensor measurement error, 2% relative distance error between the sensor and propellant, and 0.5% data acquisition system error; the overall error was approximately $\sqrt{0.02^{2}+0.02^{2}+0.005^{2}}\times 100\%=2.87\%$. Additionally, the mesh size and characterization of the propellant in the experimental and numerical simulations were included. However, the errors between the experimental and numerical simulation results were within the acceptable range, indicating that the model developed in this study was feasible for predicting the propellant. Therefore, the model and parameters could be used to calculate the impact initiation and detonation growth of the high-energy propellant at different temperatures.

## 4. Impact Response of Propellant at Different Temperatures

To further investigate the influence of thermal damage on the sensitivity of high-energy. The tests were designed and conducted to study the EFP impact test at different temperatures.

### 4.1. The EFP Impact Test at Different Temperatures

#### 4.1.1. Experimental Setup

**Figure 23:**
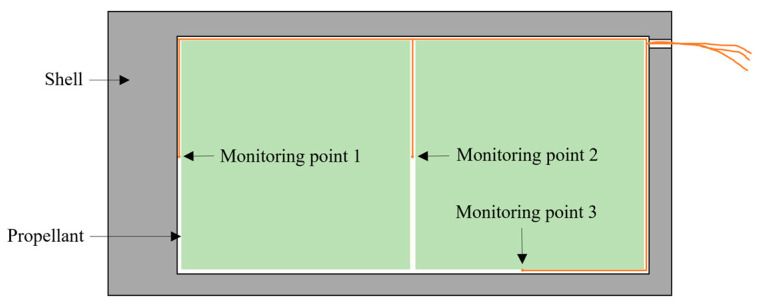
Temperature measurement point distribution at 70 °C.

**Figure 24:**
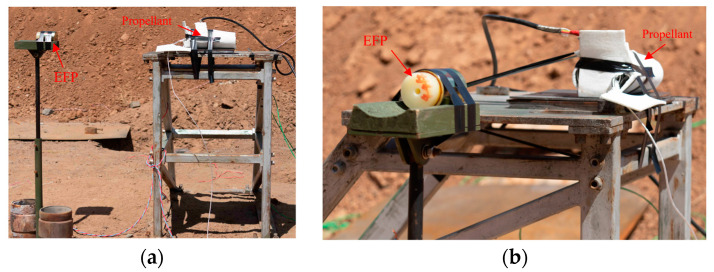
Experimental device of the EFP impact test. (**a**) Front and (**b**) side.

Figure 23 shows the temperature measurement point distribution. Three thermocouples were placed inside the propellant casing to monitor the temperatures at the top, center, and side. The experimental device is illustrated in Figure 24.

#### 4.1.2. Experimental Results

**Figure 25:**
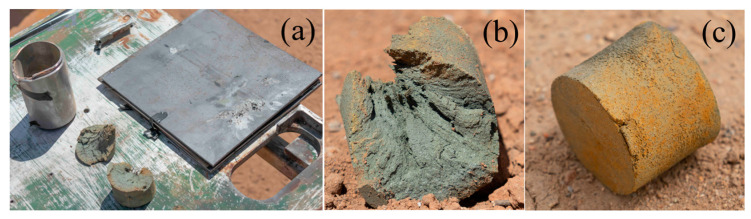
Experimental results after the EFP impact experiment at 20 °C. (**a**) Witness Plate, (**b**,**c**) propellants.

After the EFP impact experiment on N15 at 20 °C, the witness plate, shell, and propellant conditions are shown in Figure 25. It can be observed that the witness plate surface had no significant deformation, with only erosion marks at the head of the casing, resulting from a contact with a high-temperature and high-pressure liquid metal flow generated during the EFP impact. The propellant shell remained intact without severe damage. Both sections of the propellant charge were recovered. In Figure 25c, clear damage can be seen on the propellant residue caused by the EFP impact. One of the propellant charges moved about 30 m, but its structure remained intact. Based on the condition of the witness plate, casing, and propellant after the experiment, it was determined that N15 with a shell thickness of 15 mm did not react under the EFP impact at 20 °C. The hot spots generated by the heat buildup of the propellant during EFP impacts this. However, some of the energy in the shock wave is absorbed by the expansion and deformation of the propellant and the protection of the shell. The hot spot temperature did not reach the reaction temperature of the propellant. Thus, N15 with a shell thickness of 15 mm did not react under the EFP impact at 20 °C.

**Figure 26:**
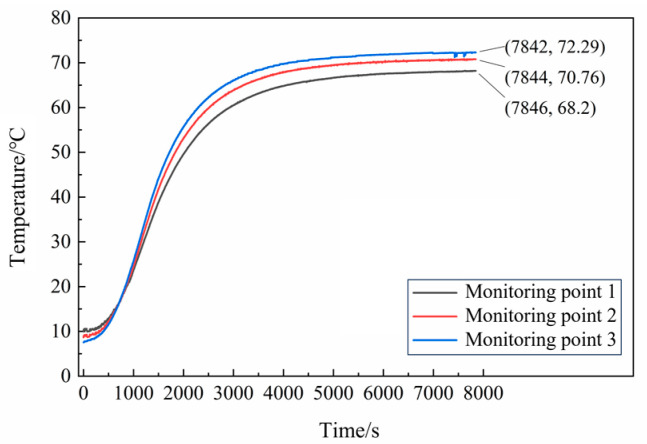
Temperature curves of N15 at 70 °C.

In the EFP impact experiment of N15 at 70 °C, the heating rate was set to 5 °C/min. The temperature inside the propellant was monitored to determine the internal temperature distribution. The temperature curves for the monitoring points are shown in Figure 26. The temperature rise inside the propellant was relatively slow. The internal temperature of the propellant stabilized after about 4300 s of heating at 70 °C. At 7200 s, the temperatures recorded by thermocouples 1, 2, and 3 were 68.01, 70.49, and 72.13 °C, respectively, and the internal temperature of the propellant satisfied the conditions for the 70 °C impact initiation test.

**Figure 27:**
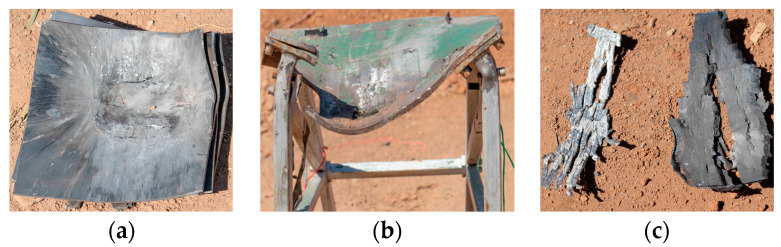
Experimental results after the EFP impact test at 70 °C. (**a**) Witness Plate, (**b**) steel support, and (**c**) shell fragments.

After the experiment, the witness plate, supports, and propellant shell can be seen in Figure 27. No residual propellant was recovered on-site. The witness plates were significantly dented. The steel supports showed severe deformation and fracture owing to a downward depression. Two fragments of the shell indicate axial tearing of the propellant shell. Based on the deformation of the witness plate, supports, and shell, it was concluded that the detonation occurred. This is mainly caused by two factors; on the one hand, the initial temperature of the explosive is increased, resulting in an increase in the chemical reaction activity of the explosive. The chemical reaction of explosives is more likely to occur under impact. On the other hand, the density of the propellant at 70 °C decreases due to the thermal expansion of the explosive particles. The propellant is more susceptible to plastic deformation when it is subjected to impact loading. The plastic deformation generates a lot of heat, and the heat and gas products are released by the decomposition of oxidizer particles when the temperature rises to a certain level. The chemical reaction of explosives is more likely to occur under impact.

In conclusion, when the thickness of the end cap was 15 mm, the unheated N15 high-energy propellant did not react, and the N15 high-energy propellant specimen reacted at a partially detonated level when it was heated to 70 °C. Under a typical high-temperature environment, the N15 high-energy propellant thermal expansion leads to a decrease in the density of the propellant and the number of pores increased, reducing the mechanical properties of the propellant; N15 impact detonation safety is significantly reduced.

### 4.2. EFP Impact Simulation of Propellant

#### 4.2.1. Computation Model

**Figure 28:**
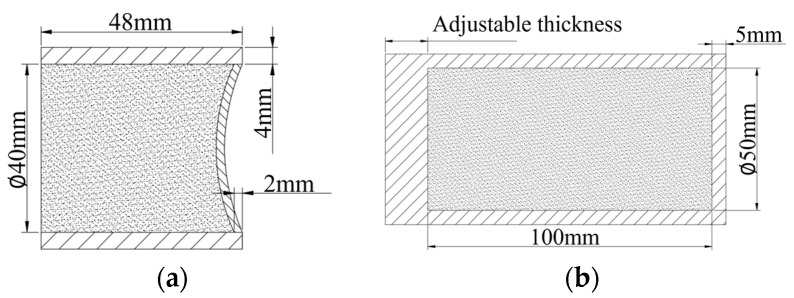
The structure of EFP and propellant. (**a**) EFP and (**b**) propellant.

The EFP structure is shown in Figure 28a, the shaped charge was 8701 explosives and the cover material was purple copper with a thickness of 2 mm. The dimensions of the N15 propellant with the shell are shown in Figure 28b, and the material of the shell is 45# steel.

#### 4.2.2. Model Parameters

The model parameters of the shaped charge and shell are shown in Table 8 and Table 9.

#### 4.2.3. Analysis of Simulation Results

**Figure 29:**
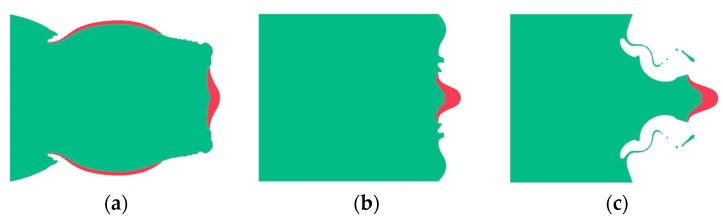
The formation process of EFP. (**a**) *t* = μs, (**b**) *t* = 40 μs, and (**c**) *t* = 83 μs.

The formation process of EFP is presented in Figure 29. After the charge detonation, under the action of the spherical detonation wave, the drug mask was crushed and flipped to form an axisymmetric, regular shape of the EFP. At t= 83 μs, the stable EFP had formed, the head velocity was 2569 m/s. The overall velocity of the EFP impacting the propellant casing was 2560 m/s.

**Figure 30:**
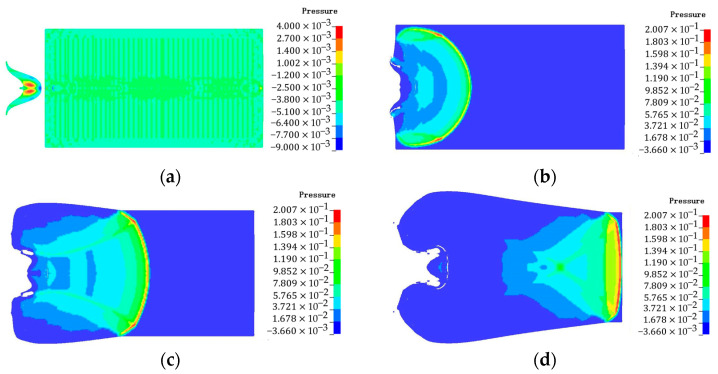
Pressure contours of N15 at 20 °C (end cover thickness: 5 mm, Unit is 100 GPa). (**a**) *t* = 198 μs, (**b**) *t* = 205 μs, (**c**) *t* = 208 μs, and (**d**) *t* = 214 μs.

When the thickness of the end cover is 5 mm, the pressure contours of N15 are shown in Figure 30. According to simulation and impact detonation theory, this process is mainly divided into three stages. Firstly, the head of the EFP impacts the steel shell at 198 μs, a plastic deformation zone and a high-pressure zone are rapidly formed around the contact area. When the depth of penetration increases, the EFP residue will spread along the bottom of the crater toward the region of strong plastic flow. The pressure wave generated by the impact of the EFP is incident into the charge. It is accompanied by complex wave system reflection and transmission phenomena. Secondly, a pressure wave of 18 GPa was formed inside the propellant at 205 μs. The pressure wave incident to the back of the shell within the charge and was accompanied by a complex wave system of reflection and transmission phenomena. The hot spots are created by the accumulation of heat from the adiabatic compression of the propellant under the action of the shock wave. When the hot spot’s temperature reaches a certain value, the pressure waves were generated by chemical reactions. Thirdly, the pressure waves propagate to unreacted areas, it enhanced the initial shock wave. When the pressure wave catches up with the impact wave, they superimpose and develop a steady detonation wave. The pressure wave propagated stably within the propellant, reaching the bottom of the propellant at 214 μs.

**Figure 31:**
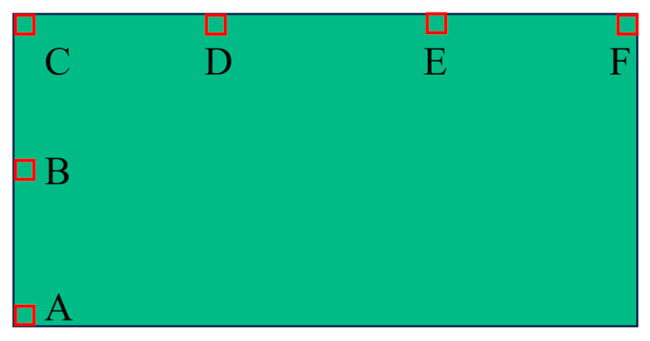
Schematic of the different locations.

**Figure 32:**
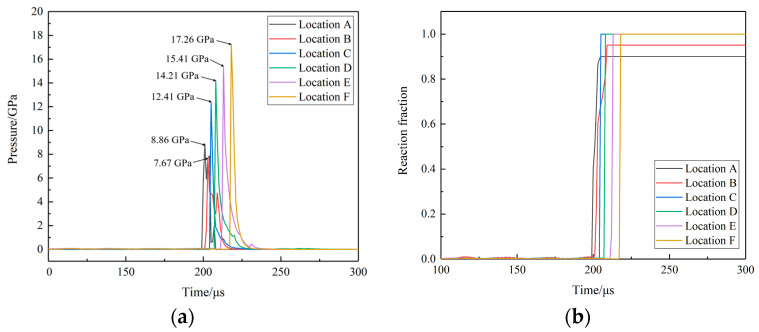
The histories of pressure and reaction fraction at different locations. (**a**) The pressure curves at different locations and (**b**) the reaction fraction curves at different locations.

Six locations, as shown in Figure 31, are selected to observe the reaction process of the propellant. The pressure curves at different locations of the N15 high-energy propellant when the thickness of the end cover is 5 mm are shown in Figure 32. The chemical reaction first occurred at positions A and B, due to the impact of the EFP. But the intensity of the external stimulus is not strong enough to make the reaction complete. Locations C, D, E, and F developed a stabilized detonation under the combined effects of the EFP impact and the shock waves generated by chemical reactions at other positions. At t = 201 μs, location A was subjected to the pressure of 8.5 GPa, which was generated by the impact of the EFP on the shell. At t = 208 μs, location A was subjected to the effect of the shockwave, which was generated by the chemical reaction in location C, which leads to the secondary pressure rise. Locations C, D, E, and F are the four observation points on the outside of the high-energy propellant pillars, and with the axial propagation of the spherical pressure wave inside the propellant, the chemical reaction begins to take place at these locations. At t = 214 μs, the pressure wave reaches the bottom of the propellant and the pressure peak at location F was 17.4 GPa.

**Figure 33:**
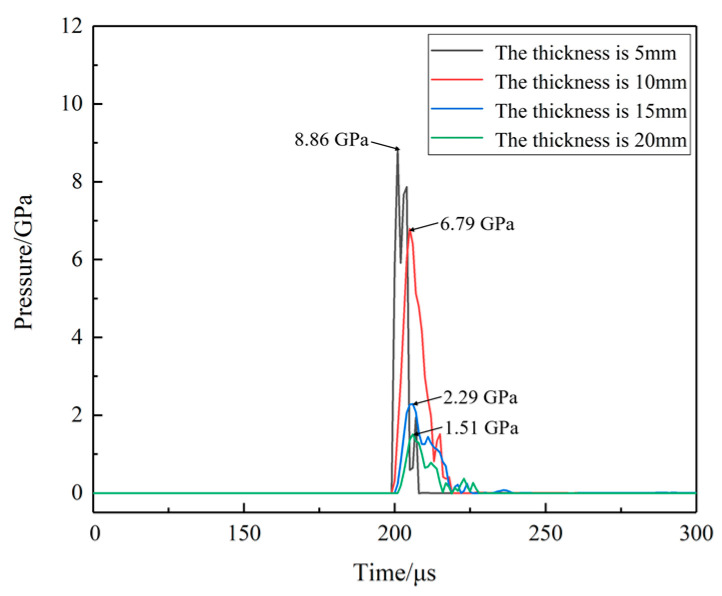
The pressure curves at location A for different shell thicknesses.

**Figure 34:**
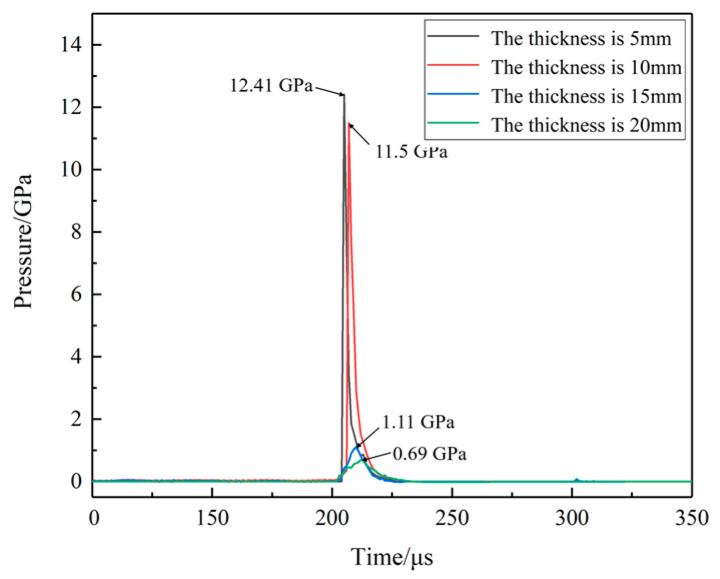
The pressure curves at location C for different shell thicknesses.

The pressure curves at location A and C for different shell thicknesses are shown in Figure 33 and Figure 34. When the thickness is 10 mm, the shock wave pressure generated at location C is larger than the pressure wave which is generated by the impact of the EFP, location C does not show a secondary pressure rise. With the increase in the shell thickness, the pressure on the propellant during the impact of EFP decreases dramatically. The peak pressure at location A is reduced from 8.5 GPa to 1.4 GPa at location A. The peak pressure at location C is reduced from 12.4 GPa to 0.8 GPa. When the thickness of the shell is 15 mm, the surface of the propellant is compressed under the initial impact, and the phenomenon of temperature increase is observed. The accumulation of heat generates a hot spot in location A. However, due to the expansion of the propellant, the protection of the shell absorbed part of the energy of the shock wave so that the hot spot stopped generating heat, the temperature of the propellant did not reach the reaction temperature, and the reaction not happen.

By adjusting the end cover thickness of the propellant casing, the threshold shell thickness for the EFP impact initiation at the initial temperatures of 20 and 70 °C for N15 was determined. The calculated results for different end cover thicknesses are listed in Table 10. As the end cover thickness increased, the reaction level of N15 changed from detonation to non-reaction. Specifically, when the end cover thickness was 15 mm, N15 heated at 20 °C did not initiate, while that heated to 70 °C underwent detonation, indicating a significant decrease in the impact safety of N15 due to the increased temperature.

## 5. Conclusions

The theoretical analysis, experimental testing, and numerical simulation were employed to systematically investigate the microstructural damage and impact initiation characteristics of the N15 propellant at different temperatures. The main conclusions are as follows:

1. The quantitative analysis of the thermal decomposition characteristics and damage features of N15 before and after the heating was conducted using TG-FTIR-MS, Micro-CT, and SEM. The heating process increased the porosity of the sample by 0.89%, mainly owing to the presence of discrete small pores in the binder matrix, rather than the formation of interconnected pores. The primary manifestation was interfacial de-bonding between the AP and the binder.
2. The cylinder test and heating Lagrange test under two typical temperatures were conducted, the detonation growth distance of N15 ranged from 6.52 to 9.84 mm, while at 70 °C, it ranged from 2.8 to 5.53 mm. The temperature elevation resulted in a shorter detonation growth distance and increased the detonation growth velocity. Genetic algorithms were used to calibrate the theoretical model parameters of N15 at 20 and 70 °C.
3. Numerical simulations and impact tests were conducted for EFP impact at different temperatures. The model parameters were validated and the critical shell thickness was determined, whose values for the EFP impact initiation were found to be 15 and 20 mm at 20 and 70 °C, respectively. The thermal damage significantly affected the impact initiation safety of N15.

In this paper, the thermal decomposition and damage characteristics of the propellant during the heating process were quantitatively analyzed. Additionally, the effects of ambient temperature on impact initiation and detonation growth of the high-energy propellant were elucidated at a mesoscopic level. The model used in this paper can describe the impact initiation characteristics of propellants at different temperatures. The results of this study can provide references for the design and optimization analysis of the impact safety of propellant. However, in order to further improve the applicability of the model, a sub-model, which includes the evolution of microvoids and microcracks, should be incorporated into the framework, to describe the effects of microstructure of propellant on hot spot formation under low-level impact.

## Figures and Tables

**Table 1:** Number of pores in N15 samples at different temperatures.

Temperature/°C	0–1 /μm^3^	1–10 /μm^3^	10–10^2^ /μm^3^	10^2^–10^3^ /μm^3^	10^3^–10^4^ /μm^3^/	10^4^–10^5^ /μm^3^	10^5^–10^6^ /μm^3^	10^6^–10^7^ /μm^3^
20	14,250	11,687	1478	137	16	5	2	0
70	5231	16,834	8934	643	43	4	4	2

**Table 2:** Specific surface area of pores at different temperatures.

Temperature/°C	Superficial Area of Pores/μm^2^	Sample Volume/μm^3^	Specific Surface Area of Pores/μm^−1^
20	4.88 × 10^5^	2.63 × 10^8^	1.86 × 10^−3^
70	1.78 × 10^6^	5.40 × 10^8^	3.29 × 10^−3^

**Table 3:** JWL equation parameters of N15.

$\rho_{0}$/(g·cm^−3^)	*A*/GPa	*B*/GPa	*R*_1_	*R*_2_	*ω*	*E*_0_/(kJ·cm^−3^)
1.829	2341.71	36.747	7.7769	1.7802	0.3205	8.67

**Table 4:** JWL equation parameters of unreacted N15.

$A_{e}$/GPa	$B_{e}$/GPa	$R_{1e}$	$R_{2e}$	$\omega_{e}$	$C_{V_{e}}$/(GPa·K^−1^)
36,943	−2.55	10.46	0.4	0.8867	2.78 × 10^−3^

**Table 5:** The peak pressures of N15 at 20 °C.

Distance/mm	Experiment/GPa	Simulation/GPa	Error
3.25	15.18	16.13	6.25%
6.52	18.17	18.21	0.22%
9.84	22.3	22.5	0.90%
13.34	22.6	22.3	1.33%

**Table 6:** The model parameters of N15 at 20 °C.

Parameter	Parameter Value	Parameter	Parameter Value	Parameter	Parameter Value
*I*/μs^−1^	4.18 × 10^10^	*G*_1_/(Mbar^−2^·μs^−1^)	346.55	*G*_2_/(Mbar^−2^·μs^−1^)	569.05
*a*	0.059	*c*	0.277	*e*	0.333
*b*	0.667	*d*	0.667	*g*	1.0
*x*	11.11	*y*	2.0	*z*	2.0

**Table 7:** The peak pressures of N15 at 70 °C.

Distance/mm	Experiment/GPa	Simulation/GPa	Error
2.80	18.3	17.8	2.73%
5.53	22.9	22.1	3.49%
8.90	22.4	23.0	2.68%

**Table 8:** Model parameters of 8701 explosive.

$\rho_{0}/$(g·cm^−3^)	$P_{CJ}$/GPa	$V_{D}$/(m·s^−1^)	*A*/GPa	*B*/GPa	*R*_1_	*R*_2_	*ω*	*E*_0_/(kJ·cm^−3^)
1.70	34.0	8390	581.4	6.801	4.1	1.0	0.35	9.0

**Table 9:** Model parameters of 45# steel.

$\rho_{0}/$(g·cm^−3^)	E/GPa	A/GPa	B/GPa	C	n	m
7.8	200	0.507	0.320	0.064	0.28	1.06

**Table 10:** Simulation results of EFP impact at different temperatures.

Thickness/mm	Temperature/°C	
	20	70
5	detonation	detonation
10	detonation	detonation
15	no initiation	detonation
20	no initiation	no initiation

## Data Availability

Data will be made available on request.
